# The Hypoxia-Inducible Factor Pathway, Prolyl Hydroxylase Domain Protein Inhibitors, and Their Roles in Bone Repair and Regeneration

**DOI:** 10.1155/2014/239356

**Published:** 2014-05-11

**Authors:** Lihong Fan, Jia Li, Zefeng Yu, Xiaoqian Dang, Kunzheng Wang

**Affiliations:** Department of Orthopedics, The Second Affiliated Hospital of Xi'an Jiaotong University, Xiwu Road, Xi'an, Shaanxi 710004, China

## Abstract

Hypoxia-inducible factors (HIFs) are oxygen-dependent transcriptional activators that play crucial roles in angiogenesis, erythropoiesis, energy metabolism, and cell fate decisions. The group of enzymes that can catalyse the hydroxylation reaction of HIF-1 is prolyl hydroxylase domain proteins (PHDs). PHD inhibitors (PHIs) activate the HIF pathway by preventing degradation of HIF-**α** via inhibiting PHDs. Osteogenesis and angiogenesis are tightly coupled during bone repair and regeneration. Numerous studies suggest that HIFs and their target gene, vascular endothelial growth factor (VEGF), are critical regulators of angiogenic-osteogenic coupling. In this brief perspective, we review current studies about the HIF pathway and its role in bone repair and regeneration, as well as the cellular and molecular mechanisms involved. Additionally, we briefly discuss the therapeutic manipulation of HIFs and VEGF in bone repair and bone tumours. This review will expand our knowledge of biology of HIFs, PHDs, PHD inhibitors, and bone regeneration, and it may also aid the design of novel therapies for accelerating bone repair and regeneration or inhibiting bone tumours.

## 1. Introduction

Hypoxia-inducible factors (HIFs) are DNA-binding transcription factors that transactivate a series of hypoxia-associated genes under hypoxic conditions to adapt to the decreased oxygen tension. Under normoxia, HIF-*α* is hydroxylated by prolyl hydroxylase domain proteins (PHDs), recognised by the ubiquitin E3 ligase, and directed to the proteasome for degradation. Under hypoxia, the catalytic activity of PHDs is inhibited. HIF-*α* accumulates in the nucleus and dimerises with HIF-*β*, and together they promote the transcription of hypoxia-response genes [1]. Considering the role of the HIF pathway in angiogenesis, erythrocytopoiesis, cell fate decisions, and cytoprotection, a large number of PHD inhibitors, especially the novel inhibitors of PHD, such as TM6008, TM6089, and FK506-binding protein 38 (FKBP38), have been found and used to activate the HIF pathway and acquire beneficial aspects of the HIF system [2, 3]. Increasing evidence suggests that the HIF pathway plays an essential role in bone regeneration and repair. The initial factor of bone regeneration is the upregulation of HIF-1*α* induced by hypoxia [4]. Resident cells, including chondrocytes and osteoblasts, sense the reduced oxygen tension via the PHDs and activate the HIFs, thereby increasing oxygen-regulated gene expression, including that of vascular endothelial growth factor (VEGF), to promote angiogenesis and osteogenesis. This review aims at discussing the advances of the HIF pathway, the relationship between angiogenesis and osteogenesis, and the role of the HIF pathway in angiogenic-osteogenic coupling. Moreover, therapeutic manipulation of the HIF pathway for bone fracture, osteoporosis, distraction osteogenesis, and bone tumour will be discussed.

## 2. Hypoxia-Inducible Factors

Hypoxia-inducible factors are DNA-binding transcription factors that interact with specific nuclear cofactors under hypoxia, and they transactivate a series of hypoxia-associated genes to trigger adaptive responses. HIFs are heterodimers composed of an *α* subunit and a *β* subunit [5]. HIF-*β* is a constitutive subunit expressed in the nucleus, and its activity is not affected by hypoxia, whereas HIF-*α* is a functional subunit, and its protein stability, subcellular localisation, and transcriptional potency are affected by oxygen levels [6]. HIFs have three members: HIF-1, HIF-2, and HIF-3, and they have the same *β* subunit but have different *α* subunits (HIF-1*α*, HIF-2*α*, and HIF-3*α*, resp.). The structure and functions of HIF-1*α* are more extensively studied than those of HIF-2*α* or HIF-3*α*. HIFs play a central role in the regulation of gene expression by oxygen. Approximately 100 target genes of HIFs have been identified, consisting of erythropoietin (EPO), vascular endothelial growth factor, hemeoxygenase-1 (HO-1), inducible nitric oxide synthase (iNOS), glucose transporter protein 1 (Glut-1), insulin-like growth factor 2 (IGF-2), endothelin 1, transferrin, and others. HIF target genes are particularly relevant to angiogenic factors, proliferation or survival factors, glucose transporters, and glycolytic enzymes [7].

Under normoxia and in the presence of Fe2+ and 2-oxoglutarate, certain conserved proline residues (Pro-402 and Pro-564 in HIF-1*α* [8, 9]; Pro-405 and Pro-531 in HIF-2*α*; Pro490 in HIF-3*α*) within the oxygen-dependent degradation domain (ODD) are hydroxylated by prolyl hydroxylase domain proteins [10–12]. Then, the hydroxylated HIF-*α* interacts with the *β*-domain of von Hippel-Lindau tumour suppressor protein (pVHL) and is subsequently ubiquitylated by the pVHL-E3 ligase complex, thereby marking HIF-*α* for degradation by the 26S proteasome [13, 14]. Under hypoxia, oxygen-dependent proteolytic destruction of the hypoxia-inducible factor-*α* subunit is abrogated [15]. HIF-*α* accumulates in the nucleus and dimerises with HIF-*β*. Together, they interact with the transcriptional coactivators p300/Creb-binding protein and form a transcriptional complex  [16, 17]. This transcriptional complex binds to hypoxia-response elements (HREs) comprising a core 5′-(A/G)CGTG-3′ consensus sequence and highly variable flanking sequences in the promoters of hypoxia-response genes  [18] and promotes the transcription of hypoxia-response genes, thereby regulating the cellular adaptive response to hypoxia  [1].

## 3. Prolyl Hydroxylase Domain Proteins

The group of enzymes that can catalyse the hydroxylation reaction of HIF-1*α* is prolyl hydroxylase domain proteins (PHDs). PHDs play a critical role in regulation of HIFs. PHDs are considered in vivo oxygen sensors insofar as they can sense the concentration of cytosolic oxygen, and they need oxygen in the form of dioxygen for catalytic activity. Under normoxia, PHDs hydroxylate two highly conserved proline residues located within the ODD recognised by the ubiquitin E3 ligase and are directed to the proteasome for degradation. Under hypoxia, PHD activity decreases due to their need for molecular oxygen as a cosubstrate. The PHD subfamily has three members: PHD1, PHD2, and PHD3. A recently characterised prolyl 4-hydroxylase possessing a transmembrane domain named PHD4 or P4H-TM was found to be the fourth member of the PHD subfamily  [19]. The investigations of PHD1–3 were more extensive compared to those of PHD4. PHDs belong to a superfamily of iron- and 2-oxoglutarate-dependent dioxygenases, meaning that molecular oxygen, 2-oxoglutarate (2-OG), and iron(II) are required for their catalytic activity. They share a well-conserved hydroxylase domain in their C-terminal halves, whereas the N-terminal halves are more variable and have poorly characterised functions among these three isoforms  [20]. They all have the ability to hydroxylate the special proline residues of HIF-*α* but differ in their substrate specificity and their distribution in tissues and cells. It was reported that PHD2 is more active on HIF-1*α* than on HIF-2*α*, whereas PHD1 and PHD3 hydroxylate HIF-2*α* more efficiently [21].

Interestingly, while PHDs regulate HIF-*α* protein stability, PHD2 and PHD3 (but not PHD1) are themselves subject to feedback upregulation by HIFs. Studies found that the PHD2 gene contains a hypoxia-response element (HRE) that can be recognised by HIF-1*α*, and the PHD3 gene contains a HRE recognised by HIF-2*α* [22, 23]. Under hypoxia, PHD2 and PHD3 are upregulated due to HIF-*α* accumulation [24–26].

## 4. Prolyl Hydroxylase Domain Protein Inhibitors

In view of the beneficial aspects of the HIF system, PHDs have been considered as therapeutic targets for anaemia and ischemia. PHD inhibitors have been designed and used to activate the HIF pathway and acquire beneficial aspects of the HIF system.

### 4.1. Nonselective PHD Inhibitors

Because PHDs require iron and 2-oxoglutarate to catalyse HIF prolyl hydroxylation, any compound that competes for endogenous iron(II) or 2-oxoglutarate can be considered a PHD inhibitor.

Deferoxamine (DFO) and cobalt chloride (CoCl_2_), an iron chelator and a competitive inhibitor of iron, respectively, are routinely used both in vitro and in vivo to inhibit PHD activity by competing for endogenous iron(II). Other iron chelators, such as ciclopirox olamine, and competitive inhibitors of iron, such as Cu2+, Zn2+, and Mn2+, are also used as PHD inhibitors [27]. However, they have effects on endogenous iron(II) and impair the activity of both PHD enzymes and other iron-dependent enzymes. Oxoglutarate analogues such as L-mimosine (L-mim), dimethyloxalylglycine (DMOG), and 3,4-dihydroxybenzoate (3,4-DHB) inhibit PHDs by competing for endogenous 2-oxoglutarate. Many studies based on cells or animals have revealed that oxoglutarate analogues can produce beneficial biological results by stabilising HIFs [28–31]. In recent years, attention has been directed towards identifying small molecule inhibitors of PHDs. Many novel small molecule inhibitors of PHDs, such as FG-2216, FG-4592, FG-4497, and GSK360A, have been recently designed by companies such as GlaxoSmithKline and FibroGen and have achieved good clinical value, especially in the treatment of anemia and ischemia [32–35].

### 4.2. Novel PHD Inhibitors

In addition to the strongly conserved catalytic site in PHDs, other domains, such as the amino or carboxyl terminal ends, which are highly variable, or the protein-protein interaction domain can also be targets for therapeutic intervention. TM6008 and TM6089, two novel PHD inhibitors, were synthesised based on the three-dimensional protein structure of human PHD2. Both of these compounds selectively inhibit PHD2 by binding to the active site within PHD2 where HIFs bind and stimulate HIF activity  [2]. The FK506-binding protein 38 (FKBP38) has been demonstrated to decrease PHD2 protein stability through interaction with N-terminal domain of PHD2 [3]. MAPK organiser 1 (Morg1), as a molecular scaffold of PHD3, also specifically interacts with the N-terminal region of PHD3  [36].

### 4.3. Untoward Effects of PHD Inhibitors

Currently available PHD inhibitors are all nonselective and nonspecific. They will certainly inhibit other 2-OG- or iron(II)-dependent oxygenases and other PHD isoforms, resulting in unwanted side effects. Moreover, recent findings have shown that, in addition to HIFs, PHDs interact with various other proteins including FKBP38 and Morg-1. Thus, treatment with PHD inhibitors has the potential to disrupt not only PHDs but also potential non-HIF substrates. VEGF, one important HIF target gene, is believed to play an important role in tumour angiogenesis and subsequent tumour growth [37]. High levels of EPO, another important HIF target gene, are closely linked to cardiovascular events and thromboembolic events. Therefore, it will be important to weigh the advantages and disadvantages when considering using PHD inhibitors as a therapy.

In addition to PHD inhibitors, many methods used to enhance the HIF pathway activity have been experimentally applied. The most common approaches used to activate HIFs are listed in Table 1, and their advantages and disadvantages are summarised.

## 5. Role of the HIF Pathway in Angiogenic-Osteogenic Coupling

Bone is a highly vascularised tissue and receives 5–20% of resting cardiac output [54, 55]. The progress of bone repair and regeneration is closely associated with that of vessel in-growth. The blood vessels supply oxygen and nutrients to the regenerating bone and mediate interactions between osteoblasts, osteocytes, osteoclasts, mesenchymal stem cells, and vascular endothelial cells.

Recently, numerous investigations have been undertaken, and it was demonstrated that HIF-1*α* and its target gene VEGF are critical regulators of angiogenic-osteogenic coupling  [56].

Hypoxia is a major driving force for angiogenesis by stabilising HIFs during endochondral bone formation [57]. Schipani et al. investigated the functions of HIF-1*α* in the cartilaginous growth plate of developing bone  [58]. They found that the developmental growth plate is hypoxic and expresses HIF-1*α* and that tissue-specific deletion of HIF-1*α* via the expression of the Col2-Cre transgene induced a massive increase in epiphyseal chondrocyte death, indicating that HIF-1*α* is required for survival in this hypoxic tissue. In addition to chondrocytes, osteoblasts also express HIF-1*α* and promote skeletal vascularisation during endochondral bone formation. Wang et al. observed the effects of gain or loss of HIF function by genetically disrupting the expression of the E3 ligase VHL or HIF-1 in osteoblasts to study the role of the HIF pathway in angiogenic-osteogenic coupling during skeletal development [59]. They found that mice that lack VHL in osteoblasts and that therefore overexpress HIFs show striking and progressive increases in bone volume. In contrast, mice lacking HIF-1 in osteoblasts show decreased bone diameter and significant reductions in osteoid volume. Interestingly, the amount of bone in these mutant mice is directly proportional to the amount of vascularisation and VEGF mRNA levels, suggesting that the regulation of bone mass may be secondary to changes in VEGF levels and angiogenesis and that HIFs and VEGF play a critical role in angiogenic-osteogenic coupling during skeletal development.

**Figure 1:**
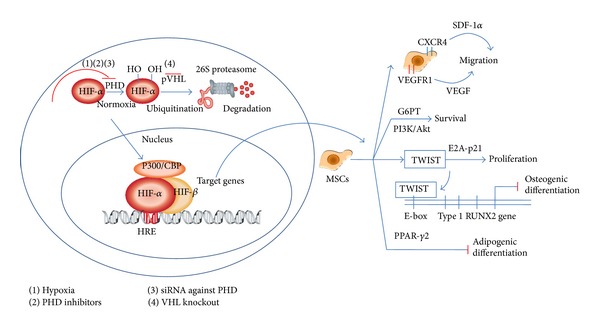
The effects of the HIF pathway on mesenchymal stem cells (MSCs). HIF-1*α* increases the proliferation of MSCs through the enhancement of TWIST expression, which downregulates the E2A-p21 pathway. The HIF pathway promotes the SDF-1*α* - and VEGF-dependent migration of MSCs by increasing the expression of the chemokine receptors CXCR4 and VEGFR1. HIFs inhibit the osteogenic and adipogenic differentiation of MSCs by decreasing the expression of RUNX2 and PPAR-*γ*2. HIFs enable MSCs to survive by activating G6PT to increase their metabolic flexibility. In summary, HIFs promote the proliferation, migration, and survival of MSCs but inhibit the osteogenic and adipogenic differentiation of MSCs.

HIFs promote angiogenesis and osteogenesis by transactivating VEGF and other HIF target genes [59]. Recent studies have demonstrated that chondrocytes, osteoblasts, and mesenchymal stem cells (MSCs) sense reduced oxygen tension via the HIF pathway and transmit signals involved in angiogenic and osteogenic gene programs during bone repair and regeneration [49]. Interestingly, the HIF pathway also has effects on osteoblasts and mesenchymal stem cells (MSCs) independent of angiogenesis. HIFs promote the proliferation, migration, and survival of MSCs, but they inhibit the osteogenic and adipogenic differentiation of MSCs [60]. Recently, HIF-1*α* has been shown to regulate MSC proliferation through the enhancement of TWIST expression, which downregulates the E2A-p21 pathway and thereby increases proliferation of MSCs [61]. The HIF pathway increases the expression of the chemokine receptors CXCR4 and VEGFR1, thereby promoting the SDF-1*α* - and VEGF-dependent migration of MSCs [62, 63]. HIFs inhibit the osteogenic and adipogenic differentiation of MSCs by decreasing the expression of RUNX2 and PPAR-*γ*2 [64–66]. It is suggested that the glucose 6-phosphate transporter (G6PT), which is activated by HIFs, enables MSCs to survive under hypoxic conditions by increasing their metabolic flexibility [67]. The effects of HIFs on MSCs are summarised in Figure 1. It has been revealed that VEGF, a target gene of HIFs, also has effects on MSC properties. VEGF promotes the proliferation, migration, and survival of MSCs, and it induces osteogenic but decreases adipogenic differentiation of MSCs [68–73]. Moreover, VEGF promotes osteoblast proliferation, survival, and migration and acts as a potent chemoattractant to induce migration in cultured osteoblasts [74, 75].

The relationship between osteoprogenitor and endothelial cells has been recently studied. Mesenchymal stem cells can provide a local environment that favours migration and formation of tubular structures of endothelial cells [76]. Moreover, mesenchymal stem cells promote the migration, extracellular matrix invasion, proliferation, and survival of endothelial cells [77]. Interestingly, endothelial cells also have effects on bone marrow stromal cells by inducing osteoblast differentiation [78].

## 6. Applications of HIF Pathway in Bone Repair and Regeneration

As the HIF pathway plays a vital role in angiogenic-osteogenic coupling, manipulation of the HIF system via pharmacological or genetic approaches is an attractive strategy for treating hypoxic-ischemic diseases, including skeletal diseases such as bone fracture.

### 6.1. Bone Fracture

Fracture, accompanied with disruption of the blood vessels, leads to activation of coagulation and formation of a haematoma that remarkably promotes angiogenesis. Simultaneously, inflammatory cells are recruited, and inflammatory signals are released to stimulate the recruitment of mesenchymal progenitors that subsequently differentiate into chondrocytes and produce a cartilaginous matrix. VEGF produced in the newly formed cartilaginous callus stimulates neoangiogenesis of the cartilage; then, the hypertrophic chondrocytes undergo apoptosis, which is followed by callus remodelling by newly recruited osteoclasts and replacement with woven bone [79, 80]. Inhibition of angiogenesis during bone repair impedes the replacement of chondrocytes with osteoblasts, resulting in the formation of fibrous rather than mineralised tissue [81].

The upregulation of HIF-1*α* induced by the interruption of oxygen and nutrient supply has been considered a primary stimulus for new bone formation [4]. Komatsu and Hadjiargyrou demonstrated for the first time that HIF-1*α* is upregulated at both transcriptional and translational levels in the femoral fracture callus during fracture repair, with peak increases on postfracture day 10 [82]. They indicated that postfracture day 10 might be a key angiogenic time point in the developing rat fracture callus. This result may offer guidance for the clinical treatment of bone fractures. VEGF is also upregulated at fracture sites [83, 84].

Numerous studies have shown that fracture repair can be promoted via manipulation of HIF pathway activity. Rios et al. found that using siRNA against PHD2 to activate the HIF-1 pathway supports bone regeneration in vivo [50]. They implanted chambers containing silk fibroin-chitosan scaffolds with siRNA against PHD2 over the periosteum. After 70 days, they found that the mean bone volume increased in vivo, suggesting that HIF-1 supports bone regeneration.

VEGF is a key regulator of angiogenesis. VEGF has been safely and efficiently applied to stimulate neovascularisation in ischemic tissues. In addition, VEGF plays an important role in bone repair and regeneration by promoting angiogenesis. Street et al. reported that treatment of mice with a soluble, neutralising VEGF receptor reduced angiogenesis, boneformation, and callus mineralisation in femoral fractures and inhibited healing of a tibial cortical bone defect, while exogenous VEGF enhanced blood vessel formation, ossification, and new bone (callus) maturation in mouse femur fractures, and it promoted bony bridging of a rabbit radius segmental gap defect [85]. Tarkka et al. confirmed that VEGF promotes bone healing by accelerating endochondral bone formation based on adenoviral-VEGF gene transfer by injecting adenoviral VEGF-A into the muscle layer surrounding the bone defect [86]. In other studies, Geiger et al.and Huang et al. also observed the same results [87, 88].

Considering that HIF degradation is regulated by PHDs, small molecule inhibitors of PHD can be used to selectively activate the HIF pathway to facilitate bone regeneration. Shen et al. directly injected DFO and DMOG, two small molecule PHD inhibitors, at fracture sites to activate HIF-1 in a stabilised murine femur fracture model [40]. They found that the vascularity increased at 14 days and the callus size increased as assessed by microCT at 28 days, suggesting that HIF activation is a viable approach to increase vascularity and boneformation following skeletal trauma. Stewart et al. activated the HIF pathway using deferoxamine combined with a biodegradable, weight-bearing scaffold to promote healing in a femoral segmental defect model [41]. Their study revealed that DFO improves angiogenesis and stiffness of bone healing in segmental defects as assessed by angiography and biomechanical testing. Zhang et al. implanted deferoxamine-loaded true bone ceramic scaffolds into 15 mm rabbit radial defects [42]. After 8 weeks, the reconstruction of bone defects was evaluated with X-ray, microCT, and histological examinations. The examinations showed considerably increasing new bone in deferoxamine-loaded true bone ceramic scaffolds, and many cavities in the new bone area were occupied by bone marrow elements and blood vessels.

### 6.2. Osteoporosis

Osteoporosis is a disease characterised by low bone mineral density and microstructural damage to bone tissue, leading to increased bone fragility and an increased risk of bone fracture. The decrease in bone mineral density is the consequence of an unbalanced bone remodelling process, with the occurrence of more bone resorption than bone formation. Osteoporosis predominantly affects postmenopausal women, and it causes great suffering in families and modern society. Current therapeutic options for the treatment of osteoporosis include antiresorptive drugs, especially bisphosphonates and more recently denosumab, as well as calcitonin and, for women, oestrogens or selective oestrogen receptor modulators. However, all these drugs have limitations in that they lead to a low turnover state, which means that bone formation also decreases with the decrease in bone-remodelling activity.

Recently, some studies have noted that HIF-1*α* can regulate the bone formation ability of osteoblasts in postmenopausal osteoporosis. Liu et al.   found that in female mice in which the HIF-1*α* gene in osteoblasts was conditionally knocked out, the trabecular number, volume, thickness, bone density, and some cytokines associated with osteogenesis and angiogenesis decreased significantly compared with wild-type mice 8 weeks after the mice were ovariectomised [89]. A similar investigation performed by Zhao et al.   showed that in female mice in which the HIF pathway in osteoblasts was activated via disruption of the HIF-degrading von Hippel-Lindau (VHL) protein, the bone structural and mechanical quality parameters did not change substantially after the mice were ovariectomised [48]. However, in the normal mice, the bone mineral density and mechanical strength decreased with deteriorated bone microarchitecture after the ovariectomy. This may offer a potential therapeutic target and options for the treatment of osteoporosis.

### 6.3. Distraction Osteogenesis

Distraction osteogenesis (DO) is a remarkable surgical method used to lengthen limbs or restore bone defects through an intramembranous process largely devoid of the formation of cartilage. Moreover, it has been used as a model for studying the cellular mechanisms involved in angiogenic-osteogenic coupling during bone repair and regeneration. During the progress of DO, intramembranous bone formation is induced by an external fixation device that applies gradual mechanical distraction across the osteotomy [90]. This procedure shows a close temporal and spatial relationship between bone regeneration and angiogenesis [4]. Thus, DO is an available method to study the role of the HIF pathway in bone repair and regeneration.

It was found that during normal DO, VEGFR1 and VEGFR2 mRNAs increase in parallel with VEGF mRNA. Interestingly, the administration of an antibody blockade of VEGFR1 and VEGFR2 resulted in decreases of both bone formation and vessel volume and number [91]. He et al. studied the expression of HIF-1*α* in a rabbit model of mandibular distraction osteogenesis by immunohistochemical and in situ hybridisation [92]. He and colleagues found that the expression of HIF-1*α* increases in the early phase but declines with the maturation of newly formed bone, suggesting that the production of HIF-1*α* in the distracted gap may contribute to new bone formation during gradual distraction of the mandible.

Pacicca et al. also investigated the expression of angiogenic factors during distraction osteogenesis [93]. The study showed that the expression of VEGF, assessed by immunohistochemical analysis, was localised at the leading edge of the distraction gap, where nascent osteogenesis occurs. They also detected mRNA expression of a wide variety of angiogenic factors by microarray analysis, including angiopoietins 1 and 2, both Tie receptors, VEGF-A and VEGF-D, VEGFR2, and neuropilin-1. They found that the expression of these factors is maximal during the phase of active distraction.

Wan et al. demonstrated that the HIF-1*α* pathway is activated during bone repair and can be manipulated genetically and pharmacologically to promote bone healing [43]. Genetically, they developed a mutant mouse model lacking pVHL in osteoblasts to activate the HIF signalling pathway. They found that genetic activation of the HIF-1*α* pathway promotes angiogenesis and enhances bone regeneration in distraction osteogenesis. The distraction gap is rapidly filled with blood vessels, and this is followed by increases in bone volume and callus strength. By contrast, in mice lacking HIF-1*α*, fewer blood vessels and less bone were observed. Pharmacologically, they used deferoxamine (DFO) and l-mimosine (l-mim), known PHD inhibitors, to activate the HIF signalling pathway, and they obtained similar results. In a similar study, Donneys et al. stated that DFO significantly increased bone structural and mechanical quality parameters such as bone volume fraction (BVF), bone mineral density (BMD), and ultimate load (UL) compared to nontreated controls during mandibular distraction osteogenesis [44].

Although PHD inhibitors have been applied in bone repair and regeneration, the PHD inhibitors used are mostly nonspecific. The application of novel PHD inhibitors such as TM6008, TM6089, and GSK360A in bone healing has not been reported. Future studies may focus on applying novel PHD inhibitors in bone regeneration and exploring novel therapeutic approaches and agents to facilitate bone repair.

### 6.4. Bone Tumours

Hypoxia is the most common phenomenon in solid tumours because of the increasing expenditure of oxygen by uncontrolled growth of the tumour cells. As important transcription factors that transactivate a series of hypoxia-associated genes to adapt to hypoxia, HIFs are also closely related to tumours. In the past two decades, the discovery of hypoxia-inducible factor-1 (HIF-1) led to a better understanding of the mechanism of tumour hypoxia. HIF-1 immortalises tumours by activating the transcription of its target genes, which regulate several biological processes including angiogenesis, cell proliferation, survival, glucose metabolism, and migration. Angiogenesis represents an essential step in tumour proliferation, expansion, and metastasis [94]. HIFs can promote the expression of a number of angiogenic factors, including VEGF, angiopoietins (ANG-1 and -2), platelet-derived growth factor-B (PDGF-B), and the TIE-2 receptor [95]. It was reported that HIF-1*α* null mutations severely retard tumour growth by reducing the expression of VEGF [96] and that overexpression of HIF-1*α* is associated with resistance to cancer chemotherapy and increased patient mortality. Thus, HIF-1 inhibitors appear to have antitumor effects; they offer a novel therapeutic strategy and are receiving increasing attention. Considering the potential antitumor effects of HIF-1 inhibitors, a number of chemical inhibitors and protein and nucleic acid inhibitors have been identified.

It was reported that the suppression of HIF-1*α* activity by trichostatin A downregulates hypoxia-response genes and hypoxia-induced angiogenesis in human osteosarcoma [97]. By using the small hairpin RNA (shRNA) technique, Wu et al. found that silencing HIF-1*α* decreased the expression of VEGF in the human osteosarcoma cell line SaOS-2 under hypoxia [98]. In another study by Wu Q et al., they investigated the inhibitory effects of small hairpin RNA targeting the HIF-1*α* gene on the growth of osteosarcoma in vitro and in vivo. It was found that cellular growth and survival activities decreased in SaOS-2 cells transfected with shRNA targeting HIF-1*α* under hypoxia, and the rates of formation and growth of xenograft tumours in mice transfected with shRNA targeting HIF-1*α* slowed significantly [99].

At present, a large number of HIF-1 inhibitors have been developed and applied in anticancer research. However, research on the antitumor effects of HIF-1 inhibitors in bone tumours is not sufficient. Future studies should focus on the effects of HIF-1 inhibitors on angiogenesis in bone tumours and their therapeutic efficacy.

## 7. Conclusion

The HIF pathway participates in many mammalian physiological processes. HIFs are involved in angiogenesis, erythropoiesis, energy metabolism, and cell fate decisions. Genetic and pharmacological manipulations of HIFs have been proven to activate the HIF pathway in vitro and in vivo and to stimulate HIF-mediated pathways even under normoxic conditions (Table 1). This approach activates the beneficial aspects of the HIF system, and the HIF pathway has been a therapeutic target for the treatment of hypoxic-ischemic diseases. Moreover, the HIF pathway plays an important role in the adaptation of tumour cells to hypoxia by activating target genes that regulate angiogenesis, cell proliferation, survival, glucose metabolism, and migration. The inhibitors of HIF-1 offer a novel method for the treatment of bone tumours.

**Figure 2:**
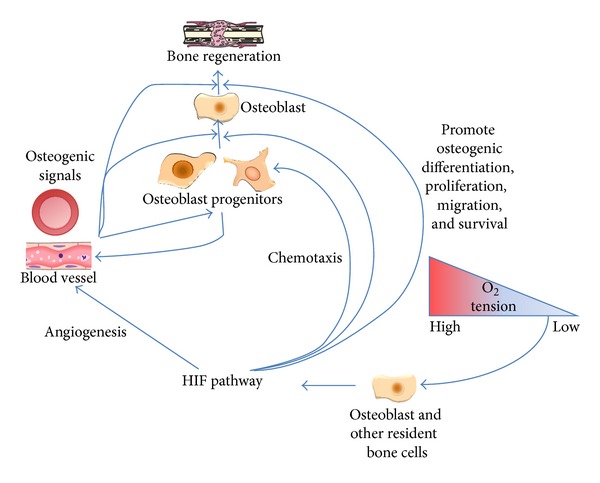
Model of the role of the HIF pathway in angiogenic-osteogenic coupling. Under hypoxia, resident cells, especially osteoblasts, sense the reduced oxygen tension via the HIF pathway; the HIF pathway is activated, leading to the upregulation of HIF-*α* and its target genes, especially VEGF. Moreover, the HIF pathway can also be activated by PHD inhibitors, siRNA against PHD, and VHL gene knockout. The accumulation of HIF-*α* and VEGF promotes bone regeneration in two ways. One way is that VEGF stimulates new blood vessel growth into bone, bringing osteogenic signals and more osteoblast progenitors, which mature and form new bone. The other is that HIF-*α* and VEGF function in an autocrine or paracrine mode to directly promote the chemotaxis of osteoblast progenitors and induce osteogenic differentiation, migration, and proliferation, accelerating bone mineralisation.

Increasing evidence suggests that the HIF pathway plays a critical role in angiogenic-osteogenic coupling during bone repair and regeneration. Indeed, the HIF pathway has been used as an approach to accelerate bone healing following injury. Considering the experimental observations reviewed in this paper, we present an angiogenic-osteogenic coupling model that describes the procedure from the sensing of reduced oxygen and nutrient availability by resident bone cells, the initiation of angiogenesis, to ultimate bone repair (Figure 2). First, resident cells such as chondrocytes, osteocytes, and osteoblasts sense reduced oxygen tension via the HIF pathway. Then, the HIF pathway is activated, and both HIFs and their target genes including VEGF are upregulated. Finally, the HIFs and VEGF promote bone regeneration via at least one of two mechanisms. One of the two mechanisms is that VEGF functions to stimulate new blood vessel formation and invasion into bone. The newly formed blood vessels deliver chemical signals that induce bone formation and carry more osteoblast progenitors that subsequently mature and form new bone. Additionally, the endothelial cells of the newly formed blood vessels can induce osteoblast differentiation of bone marrow mesenchymal stem cells. The other mechanism is that the HIF pathway may function in an autocrine or paracrine mode to directly stimulate mesenchymal stem cell and osteoblast differentiation, proliferation, and migration independent of angiogenesis, thereby accelerating bone mineralisation. This may indirectly induce the formation of blood vessels by promoting the migration, extracellular matrix invasion, proliferation, and survival of endothelial cells in view of the effects of mesenchymal stem cells on endothelial cells.

Much work needs to be done in the future to further elucidate the cellular and molecular mechanisms of angiogenic-osteogenic coupling. This work may lead to a better understanding of the precise communication between angiogenesis and osteogenesis and provide novel methods to accelerate bone repair and regeneration or inhibit the growth of bone tumours.

## Figures and Tables

**Table 1:** Approaches to HIF signal enhancement.

Approach/agent	Remarks	Applications/reference
Chemical inhibition of PHDs by hypoxia-mimetics	Operate conveniently	
Iron chelators or iron competitive inhibitors	Nonspecific; unwanted effects on endogenous iron	
DFO		[38–44]
CoCl_2_		[45]
Oxoglutarate analogues	Nonspecific	
DMOG		[40]
L-Mimosine		[43]
EDHB		[46]
FG-2216		[47]
GSK360A		[35]
Genetic techniques	Complex procedures; biotic security	
Von-Hippel-Lindau knockout	Nonspecific	[48, 49]
PHD siRNA transfection	PHD-specific	
PHD2 siRNA		[50, 51]
Constitutively active HIF-*α* transgenes	Organ-specific	[52, 53]

DFO: deferoxamine; CoCl_2_: cobalt chloride; DMOG: dimethyloxalylglycine; EDHB: ethyl-3-4-dihydroxybenzoate; FG: fibrogen; GSK: GlaxoSmithKline.

## Abbreviations

HIFs: Hypoxia-inducible factors
PHDs: Prolyl hydroxylase domain proteins
VEGF: Vascular endothelial growth factor
MSCs: Mesenchymal stem cells
FKBP38: FK506-binding protein 38
bHLH-PAS: The basic Helix-Loop-Helix PER-ARNT-SIM
EPO: Erythropoietin
HO-1: Hemeoxygenase-1
iNOS: Inducible nitric oxide synthase
Glut-1: The glucose transporter protein 1
IGF-2: Insulin-like growth factor 2
ODD: Oxygen dependent degradation domain
VHL: Von Hippel-Lindau tumor suppressor protein
FIH: Factor inhibiting HIF
C-TAD: C-Terminal transactivation domains
HREs: Hypoxia-response elements
2-OG: 2-Oxoglutarate
DFO: Deferoxamine
CoCl_2_: Cobalt chloride
L-mim: L-Mimosine
DMOG: Dimethyloxalylglycine
3,4-DHB: 3,4-Dihydroxybenzoate
Morg1: MAPK organizer 1
OS9: Osteosarcoma amplified 9
ING4: The tumor suppressor gene inhibitor of growth family member 4
IKK: IkB kinase-
EDHB: Ethyl-3-4-dihydroxybenzoate
FG: Fibrogen
GSK: GlaxoSmithKline.
